# Improving the Dietary Vitamin A Content of Rural Communities in South Africa by Replacing Non-Biofortified White Maize and Sweet Potato with Biofortified Maize and Sweet Potato in Traditional Dishes

**DOI:** 10.3390/nu11061198

**Published:** 2019-05-28

**Authors:** Laurencia Govender, Kirthee Pillay, Muthulisi Siwela, Albert Thembinkosi Modi, Tafadzwanashe Mabhaudhi

**Affiliations:** 1 Dietetics and Human Nutrition, School of Agricultural, Earth and Environmental Sciences, University of KwaZulu-Natal, Private Bag X01, Scottsville 3209, Pietermaritzburg 3201, South Africa; GovenderL3@ukzn.ac.za (L.G.); pillayk@ukzn.ac.za (K.P.); 2 Centre for Transformative Agricultural and Food Systems, School of Agricultural, Earth and Environmental Sciences, University of KwaZulu-Natal, Private Bag X01, Scottsville 3209, Pietermaritzburg 3201, South Africa; ModiAT@ukzn.ac.za (A.T.M.); mabhaudhi@ukzn.ac.za (T.M.)

**Keywords:** food and nutrition insecurity, nutrients, provitamin A-biofortified foods, bambara groundnut, orange-fleshed sweet potato, indigenous foods

## Abstract

Biofortification of staple crops has a potential for addressing micronutrient deficiencies, such as vitamin A deficiency (VAD), which are prevalent in South Africa. The poor acceptability of provitamin A (PVA)-biofortified foods could be improved by combining them with other food items to produce modified traditional dishes. The nutritional composition of the dishes could also be improved by the modification. The study aimed to investigate the effect of replacing white maize and cream-fleshed sweet potato (CFSP)] with PVA-biofortified maize and orange-fleshed sweet potato (OFSP) on the nutritional composition of South African traditional dishes. The protein, fibre, total mineral (ash), lysine, and iron concentrations of the PVA maize *phutu* (traditional porridge) composite dishes (control), were not significantly different (*P* > 0.05) from those of white maize *phutu* composite dishes. However, the PVA concentration of PVA maize *phutu* composite dishes was higher than that of the white *phutu* composite dishes (*P* > 0.05). The OFSP had a significantly lower protein concentration, but a significantly higher (*P* > 0.05) fibre, ash, lysine, isoleucine, leucine, and PVA concentration, relative to the CFSP. The findings indicate that composite dishes in which white maize is replaced with PVA-biofortified maize, and switching over from CFSP to OFSP, would contribute to combating VAD in South Africa, and in other developing counties.

## 1. Introduction

Biofortification is a process that improves the nutrient content of crops through plant breeding or recombinant DNA technology (rDNA) [1]. Biofortified crops could play a vital role in improving the nutritional status of vulnerable population groups, where supplementation, conventional fortification, or dietary diversity are limited or problematic to implement [1]. HarvestPlus, a global Challenge Programme, aims to reduce micronutrient malnutrition by developing crops that are higher in vitamin A, iron, and zinc, and have selected seven crops for biofortification, namely: beans *(Phaseolus vulgaris)*, maize (*Zea mays*), pearl millet (*Pennisetum glaucum*), wheat *(Triticum aestivum)*, cassava (*Manihot esculenta*), sweet potato (*Ipomoea batatas*), and rice (*Oryza sativa*). Of these crops, cassava, maize, and sweet potato have been selected for provitamin A (PVA)-biofortification [1,2]. The current PVA breeding targets for maize and sweet potato are 15 µg/g and 32 µg/g, respectively [3,4]. White maize and cream-fleshed sweet potato (CFSP) are two commonly grown and consumed food items in South Africa [5,6], and, therefore, are ideal for PVA-biofortification [5,6,7]. The two crops are deficient in PVA carotenoids, the precursors of vitamin A found in plants [6,7]. This could partly explain the slow improvement in the vitamin A status of the South African population, especially children [8].

Vitamin A is an essential micronutrient that has several physiological roles including immunity, vision, and protein synthesis [9]. In South Africa, between 1994 and 2005, the number of children with vitamin A deficiency (VAD) increased from 33.3% to 63.6% [10,11]. Although, the results from the SANHANES-1 study of 2012 showed a decrease in VAD prevalence, the prevalence of VAD is still high (43.6%) [8]. Biofortification could be used as a complementary strategy to reduce VAD in vulnerable population groups. However, PVA-biofortified foods, especially maize, have been found less acceptable compared to their non-PVA-biofortified counterparts [12,13,14]. This has been attributed to the unfamiliar sensory attributes imparted by carotenoid pigments present in PVA-biofortified foods [12,13,14,15,16]. Consumer acceptability of PVA-biofortified foods could be improved by combining them with other commonly consumed food items (plant or animal food sources) as they could mask the undesirable sensory properties of the PVA-biofortified foods. The only published study that investigated the acceptance of combining PVA-biofortified foods reported improvement in consumer acceptability of PVA-maize porridge when it was combined with chicken stew [17]. Unlike biofortified maize, orange-fleshed sweet potato (OFSP) is well accepted by consumers [18,19,20,21,22], and, therefore, there are fewer challenges of consumer acceptability of foods containing OFSP.

Apart from increasing the PVA content of popular vitamin A-deficient traditional and indigenous plant-based dishes of KwaZulu-Natal (KZN), PVA-biofortified maize and OFSP are also likely to affect the concentration of other nutrients in the dishes. The study objective was to determine the effect of replacing white maize and CFSP with PVA-biofortified maize and OFSP on the nutritional composition of traditional dishes of KZN province of South Africa. Traditional dishes are food types that, over a long time, have been associated with specific population group/s residing in a defined geographic location, and consumption of the dishes is passed down through generations [23]. The food types studied were *phutu* (crumbly maize porridge) prepared with white maize meal and PVA-biofortified maize meal, served with either curried chicken, curried cabbage, or curried bambara groundnut, and, separately, boiled sweet potato (CFSP and OFSP). 

## 2. Materials and Methods 

### 2.1. Plant Materials

Dried (about 10% moisture) grain of two maize varieties, a PVA-biofortified variety (PVA A) and white variety (WE-3172) (control), were used in this study. Plant breeders at the University of KwaZulu-Natal (UKZN) produced the maize grains. Orange maize inbred lines were developed through pedigree breeding [24]. The maize varieties were grown at UKZN’s Ukulinga Research Farm, Pietermaritzburg, South Africa. Standard cultural practices for maize production were followed. The maize was harvested manually and left to dry under ambient conditions (±25 °C) for 21 days at the Ukulinga Research Farm. The maize was then threshed by hand and the grain was stored in a cold room (approx. +4 °C) at Ukulinga Research Farm until it was required for the research. A grain cleaner (R.G Garvie and sons, Agricultural Engineers, Aberdeen, Scotland, UK) was used to clean the maize grains. After cleaning the grains, moisture was adjusted to 15% (*w*/*v*). A hammer roller (Zhauns Business Opportunities & Engineering, Cape Town, South Africa) was then used to mill the maize grains. This maize meal passed through a 1-mm hammer mill screen. 

The OFSP (A45) and CFSP (A40) genotypes were developed by controlled pollination. The female and male parent were known. The parents of A40 were Merikan x Yan Shu 1, and of A45 were Excel x Xushu 18. They were among the progeny of the first set of crosses conducted among elite parental lines imported from CIP, Peru. This first series of crosses were referred to as the “A” series, and proved the most successful with seven crosses being released and grown by small and large-scale farmers [25,26]. 

The bambara groundnut landrace was purchased from Umvoti beans. The bambara groundnut landraces were stored at room temperature in an airtight container. White cabbage was purchased from a local (Pietermaritzburg) supermarket for the study.

### 2.2. Methods

#### 2.2.1. Preparation of Food Products 

*Phutu* was prepared from the maize meal of the two maize varieties (Supplementary Material S1). The *phutu* was served with curried cabbage (Supplementary Material S2), curried chicken (Supplementary Material S3), and curried bambara groundnut (Supplementary Material S4). *Phutu* served with either curried cabbage or chicken were selected because they are popular traditional dishes in KZN. The *phutu* and bambara groundnut curry was selected as a plant-based alternative to animal food sources (chicken) that are usually combined with *phutu*. The two varieties of sweet potato (Supplementary Material S5) were boiled and served as stand-alone dishes, i.e. they were not composited with other food items.

#### 2.2.2. Nutritional Composition 

The nutritional composition of uncooked and cooked food samples was determined using standard or referenced methods. Before nutritional analysis, raw (uncooked) and cooked food samples with a high moisture content (all cooked samples, as well as uncooked PVA-biofortified and white maize meal) were freeze-dried. Two replicates of each sample were analysed.

***Protein.***

The protein content of the samples was measured with a LECO Truspec Nitrogen Analyser (LECO Corporation, St Joseph, Michigan, USA) using the AOAC official method 990.03 [27]. Controls and samples were measured in duplicate, and placed into a combustion chamber at 950 °C with an autoloader. The following equation [27] was used to calculate the percentage of protein:

(1) % crude potein=% N×6.25

***Fat.***

The fat content of the samples was determined following the Soxhlet procedure. The Büchi 810 Soxhlet Fat extractor (Büchi, Flawil, Switzerland) was used for the analysis according to the AOAC Official Method 920.39 [24]. Petroleum ether was used for extraction, and the percentage of crude fat was calculated using the following equation [27].

(2) $$\%\;crude\;fat=\frac{beaker+fat-beaker\times 100}{sample\;mass}$$

***Moisture.***

The moisture content of the samples was measured using the AOAC Official Method 934.01 [27]. The samples were dried at 95°C for 72 h in an air-circulated oven. Thereafter, the weight loss of the samples were used to calculate the moisture content. The following equation [27] was used to calculate the moisture content:

(3) $$\%\;moisture=\frac{\left(mass\;of\;the\;sample+dish)-(mass\;of\;sample\;after\;drying\right)}{\left(mass\;of\;the\;sample+dish)-(mass\;of\;petri\;dish\;without\;the\;lid\right)}\times 100$$

***Total mineral content (ash).***

The total mineral content of the samples was determined using the AOAC Official method 942.05 [24]. The samples were weighed and placed in a furnace at 550 °C for 24 h. The minerals remained as a residue of ash in the crucibles after the volatilisation of the organic matter from the samples. The following equation [27] was used to determine the percentage of ash:

(4) $$\%\;ash=\frac{\left(mass\;of\;the\;sample+crucible\;after\;ashing)-(mass\;of\;pre-dried\;crucile\right)}{\left(mass\;of\;sample+crucile)-(mass\;of\;pre-dried\;crucile\right)}\times 100$$

***Fibre.***

Fibre was determined as neutral detergent fibre (NDF) using the van Soest method [28,29,30]. The sample was weighed using an analytical balance and 0.5 g was added into a scintered glass crucible (34 × 2.8 mm, porosity 2). Neutral detergent solution (NDS) (50 mL) and a marble/buffer beads were added to the glass crucible holder. The NDS was prepared with 124 g ethylene diamine tetra-acetic acid, 45.3 g disodium tetraborate, 200 g sodium lauryl sulphate, 67 mL 2-ethoxy ethanol and 30.4 g disodium hydrogen phosphate. Thereafter, the crucible containing the sample was placed into the glass crucible holder. The crucible holder with crucible, sample and NDS were placed into a digestive block set at 110 °C. Subsequently, 1 mL of termamyl (α-amylase) was added and covered with stoppers. After 1 hour 10 min, the glass crucible holder containing the crucible, sample, and NDS was removed and placed into the glass crucible holder rack. Next, the glass crucible was removed and placed on a draining rack. The sample was then suctioned in a filtration unit which was connected to a vacuum system and washed three times with boiling water. The sample and sides of the crucible were rinsed with acetone. Thereafter, samples were placed in a drying oven set that was maintained at 105 °C for at least 4 h. The sample was then removed from the oven and placed in a desiccator to cool, the crucible was weighed and the following equation was used to calculate the NDF of the sample [31]:

(5) $$\%\;NDF=\frac{\left(crucible+dry\;residue)-(crucible+ash\right)}{sample\;mass}\times 100$$

***Amino Acids.***

Amino acids were analysed using the central analytical facilities (CAF) HCI hydrolysis method [32]. The freeze-dried sample was added to a glass vial and 6 N HCl was added. Thereafter, the vial was flushed with argon or nitrogen gas to eliminate oxygen, before the lid was closed. The vial was placed in an oven at 110 °C for 18–24 h. The vial was removed from the oven and allowed to cool. The hydrolysate was filtered using centrifuge tube filters (Corning^®^ Costar^®^ Spin-X tubes, Sigma-Aldrich, St. Louis, MO, USA). The filtrate was transferred to Eppendorf tubes and allowed to dry using a speed vac and thereafter reconstituted in borate buffer for derivatization. The borate buffer was transferred into a 200 µL glass insert in a 2 mL glass vial, and 10 µL of either standard solution or diluted sample was added. The 6-aminoquinolyl-*N*-hydroxysccinimidyl carbamate (AQC) reagent was added, and then the vial was placed in a vortex to ensure that the sample was mixed properly. The vial was then placed in an oven at 55 °C for 10 min, and then loaded into the autosampler tray for analysis. An H-class Waters Acquity ultra performance liquid chromatograph (UPLC) linked to a Waters photodiode array detector (Waters, Milford, MA, USA), was used for high-resolution UPLC-UV analysis. Separation was achieved on an Acquity UPLC BEH C18 (2.1 × 150 mm; 1.7 µm particle size) column at 60 °C and a flow rate of 0.4 ml/min. Data were collected at a wavelength of 254 nm. An injection volume of 1 µL was used, and gradient separation was performed using Solvents A and B from the Waters Accutag kit.

***Mineral elements.***

Calcium, phosphorous, iron, and zinc, were analysed using the Agricultural Laboratory Association of Southern Africa (ALASA) Method 6.1.1 [33]. The first step of this process was to freeze dry the samples in a freeze drier (Edwards, High vacuum international, Sussex, England). Samples were ashed for 24 h at 550 °C in a furnace. The samples were dissolved in HCl and then HNO3 was added. The samples were analysed using an atomic absorption spectrophotometer. Calcium and phosphorous were determined using the Analytik Jena Spekol 1300 spectrophotometer (Analytik Jena AG, Achtung, Germany). Iron was determined with the Varian SpectrAA atomic absorption spectrophotometer (Varian Australia Pty Ltd, Mulgrave, Victoria), and zinc with the GBC 905AA spectrophotometer (GBC Scientific Equipment Pty Ltd., Dandenong, Victoria, Australia).

***Provitamin A.***

The provitamin A content of the food samples was determined by high-performance liquid chromatography (HPLC), according to the procedures described by [34]. 

#### 2.2.3. Statistical Analysis

Nutritional composition data were analysed using the Statistical Package for Social Science (SPSS version 25.0 SPSS Inc, Chicago, IL, USA). Mean values and standard deviations were determined for all duplicate measurements for 13 cooked and uncooked samples. The Kruskal Wallis non-parametric test was used to determine if there were significant differences in nutritional composition across uncooked and cooked food products. Where significant differences were identified, the Mann-Whitney U test was used to determine the specific differences. The Mann-Whitney U test was also used to determine significant differences in nutritional composition across the two sweet potato varieties. Significance was measured at the 5% level throughout. 

## 3. Results

### 3.1. Proximate Composition 

The concentration of all the nutrients analysed differed significantly (Kruskal Wallis test: *P* < 0.05) across the 13 food samples (Table 1). Following further analysis with the Mann-Whitney U test, specific differences and are described below.

The protein concentration of PVA-biofortified maize flour/flour (8.68 g/100 g) was not significantly different (*P* > 0.05) from that of white maize meal/flour (10.22 g/100 g) (Table 1). Provitamin A-biofortified *phutu* (8.74 g/100 g) had a significantly (*P* < 0.05) lower protein concentration than that of chicken curry, but was not significantly different (*P* > 0.05) from the protein concentration of cabbage curry and bambara groundnut curry. The protein content of chicken curry (71.23 g/100 g) was approximately quintuple that of cabbage curry (13.04 g/100 g) and approximately quadruple that of bambara groundnut curry (17.00 g/100g) (*P* < 0.05). Provitamin A-biofortified *phutu* served with either curried cabbage, chicken or bambara groundnut did not have a significantly different (*P* > 0.05) protein concentration in comparison to white *phutu* served with either curried cabbage, chicken or bambara groundnut. 

When comparing the three PVA biofortified composite dishes, there were no significant differences (*P* > 0.05) in the protein concentration. 

The fibre concentration of PVA-biofortified maize/flour (13.53 g/100 g) was not significantly different (*P* > 0.05) from that of white maize meal/flour (control) (5.44 g/100 g) (Table 1). Replacing white maize *phutu* (control) with PVA *phutu* in the composite dishes had no effect on the fibre concentration of PVA *phutu* and chicken curry (*P* > 0.05); PVA *phutu* and cabbage curry (*P* > 0.05); or *phutu* and bambara groundnut curry (*P* > 0.05). A similar fibre concentration was observed in the three composite dishes containing PVA-biofortified *phutu* (*P* > 0.05).

The total mineral content (ash) of white maize meal/flour (control) (1.21 mg/100 g) was not significantly different (*P* > 0.05) from that of PVA-biofortified maize meal/flour (1.35 mg/100 g) (Table 1). The ash content of PVA-biofortified *phutu* (2.64 mg/100 g) was significantly lower (*P* < 0.05) than cabbage curry (11.19 mg/100 g), chicken curry (14.15 mg/100 g) (*P* < 0.05) and bambara groundnut curry (8.00 mg/100 g) (*P* < 0.05). Chicken curry (14.15 mg/100 g) had a significantly higher ash content compared to curried cabbage or bambara groundnut (*P* < 0.05). However, the ash content did not change significantly (*P* > 0.05) when white *phutu* was replaced with PVA *phutu* in the three composite dishes. Orange-fleshed sweet potato had a significantly (*P* < 0.05) lower protein concentration relative to the CFSP, but a significantly higher (*P* < 0.05) fibre and total mineral concentration (ash) (Table 2).

### 3.2. Amino Acid Content 

Results from the Krusal Wallis test showed that the concentration of the essential amino acids histidine, lysine, and phenylalanine (Table 3) differed significantly (*P* < 0.05) across the 13 food samples analysed (Table 4). Further analysis with the Mann-Whitney U test revealed specific differences in nutrient concentrations, which are described below.

Lysine concentration in white maize meal/flour (control) (0.40 g/100 g) was not significantly different (*P* < 0.05) from the lysine concentration in PVA-biofortified maize meal/flour (0.36 g/100 g). There was no significant difference (*P* < 0.05) in lysine concentration between PVA-biofortified *phutu* and curried cabbage (0.59 g/100 g); and bambara groundnut (1.80 g/100 g). However, the lysine content was 11.53 g/100 g higher (*P* < 0.05) in chicken curry than in PVA-biofortified flour. Chicken curry had a 11.22 g/100 g and 10.01 g/100 g higher (*P* < 0.05) lysine concentration in comparison to cabbage curry and bambara groundnut curry, respectively. Although not statistically significant (*P* < 0.05), the mean lysine concentration was higher in bambara groundnut curry than in cabbage curry. The lysine concentration did not change (*P* < 0.05) when white *phutu* (control) was replaced with PVA-biofortified *phutu* in all three composite dishes. When comparing the three composite dishes containing PVA-biofortified *phutu*, it was evident that there was no significant difference (*P* < 0.05) in the lysine concentration.

Orange-fleshed sweet potato had an improved (*P* < 0.05) lysine, isoleucine, leucine, aspartate, glutamate and alanine concentration but a lower phenylalanine concentration than CFSP (Table 5 and Table 6).

### 3.3. Mineral Composition 

Results from the Kruskal Wallis test showed that the concentration of selected mineral elements analysed differed significantly (*P* < 0.05) across all the 13 food samples, except zinc concentration, for which no significant difference (*P* < 0.05) was found (Table 7). The Mann-Whitney U test showed specific differences were identified and are described below. 

Iron concentration in white maize meal/flour (control) was not significantly different (*P* < 0.05) from iron concentration in PVA-biofortified maize meal/flour. PVA-biofortified *phutu* had a 96.8 mg/100 g lower (*P* < 0.05) iron concentration compared with chicken curry. The iron concentration of chicken curry (98.50 mg/100 g) was significantly (*P* < 0.05) higher than that of cabbage curry and bambara groundnut curry. The iron concentration did not change significantly (*P* < 0.05) when white *phutu* was replaced with PVA *phutu* in the three composite dishes. A similar iron concentration (*P* < 0.05) was found in all three composite dishes containing PVA-biofortified *phutu.*

The zinc concentration of CFSP was significantly (*P* < 0.05) higher than that of OFSP, but the CFSP had a significantly (*P* < 0.05) higher iron concentration than OFSP (Table 8).

### 3.4. Provitamin A (PVA) Composition

Results from the Kruskal Wallis test showed that the concentration of PVA analysed differed significantly (*P* < 0.05) across the 13 food samples analysed (Table 9), and the Mann-Whitney U test showed specific differences in nutrient concentration as described below.

Provitamin A-biofortified maize meal/flour had a much higher (*P* < 0.05) PVA carotenoid concentration (1.43 µg/g) than white maize meal/flour (control) (0.62 µg/g). The PVA-biofortified *phutu* had a 0.37 µg/g, 1.13 µg/g and 0.71 µg/g higher (*P* < 0.05) PVA carotenoid concentration than cabbage curry, chicken curry, and bambara groundnut curry, respectively. Both cabbage curry and bambara groundnut curry had a significantly higher (*P* < 0.05) PVA carotenoid concentration than chicken curry. The composite dish that comprised of PVA *phutu* and chicken curry (1.12 µg/g) had a significantly higher (*P* < 0.05) PVA carotenoid concentration than the composite dish of white *phutu* and chicken curry (control) (0.60 µg/g). Combining cabbage curry, which had appreciable PVA concentration (0.97 µg/g), with either white *phutu* or PVA *phutu* resulted in a nonsignificant (*P* < 0.05) PVA concentration of the two composite dishes. The PVA carotenoid concentration increased (*P* < 0.05) by 0.36 µg/g when white *phutu* was replaced with PVA *phutu* in the composite dish containing bambara groundnut curry. 

The PVA carotenoid concentration of the OFSP (55.84 µg/g DW) was much higher (*P* < 0.05) than that of the CFSP (0.77 µg/g DW) (control) (Table 10).

## 4. Discussion

Globally, about two billion people experience micronutrient deficiency, mainly because their diets are of very limited diversity [35,36]. Undernutrition, particularly protein-energy malnutrition and micronutrient deficiencies, is more prevalent in developing regions, especially sub-Saharan Africa, where a significant proportion of the population groups are poor and food insecure [11,12,13,14,35,36,37]. The poor communities cannot afford a nutritious, diversified diet; they rely heavily on monotonous diets of starchy staples, which are generally low in essential nutrients, including micronutrients and protein [38,39,40]. Among the micronutrient deficiencies, VAD as well as iron and zinc deficiencies, are generally leading [41]. This emphasises the need to provide an affordable alternative source of essential nutrients, such as vitamin A, zinc, iron, and protein. Biofortified staple crops, which are being developed such that they contain much higher concentrations of target nutrients, like vitamins and minerals, compared to the corresponding non-biofortified crops, if consumed regularly, would result in significant improvements in human health and nutrition [42]. 

As is the case with most of the countries in the sub-Saharan African region, micronutrient deficiencies, especially VAD, are a significant problem in South Africa [8,10,11]. The KZN province has the largest proportion of poor households in South Africa, and has been one of the poorest provinces in South Africa since 2011 [43,44]. Many of these impoverished communities are unable to purchase foods that form a diversified diet, and end up consuming mainly starch-based food [36]. A basic food basket comprising 28 items in South Africa costs about US$50, which is unaffordable to most rural population groups [45]. Poor households are at a high risk of malnutrition, as they cannot afford a diversified diet [46,47]. Increasing the concentration of provitamin A in staple crops through biofortification is a promising strategy for contribution to combating VAD. The results of this study are encouraging, as they indicate that PVA-biofortified maize contained a much higher PVA carotenoid concentration compared to white maize (control), which is consistent with previous studies [48]. 

It appears that, currently, there are no published data on the nutritional composition of composite dishes containing PVA-biofortified maize food products like *phutu*. However, when either cabbage curry, chicken curry, or bambara groundnut curry were combined with PVA maize *phutu*, the PVA carotenoid concentration of the composite dishes was higher than that of the corresponding composite dishes containing white maize (controls). The results indicate that composite dishes in which PVA-biofortified maize *phutu* is combined with other commonly consumed food items would be suitable carriers of provitamin A for delivery to the target population groups, such as the poor, rural communities of KZN. In the current study, bambara groundnut was included in the dishes containing maize, because it could be used as an affordable protein source, which has the added advantage of thriving in the predominantly harsh agro-ecological conditions of most of the marginal rural areas of sub-Saharan African countries, including the rural areas of KZN [49]. Previous consumer acceptability studies conducted on bambara groundnut showed promising results, but the studies investigated bambara groundnut food types different from that of the current study [50,51,52]. In terms of nutritional composition, the composite dish consisting of PVA-biofortified *phutu* and bambara groundnut curry shows a potential for improving the vitamin A and protein status of vulnerable population groups, but the acceptability of such dishes to the target population groups should be investigated, because bambara groundnut is not a familiar food item to the majority of South Africans [53].

While it is important to note that PVA carotenoids must be converted into vitamin A, which then can be used by the human body [54], a study conducted by Palmer et al. found that there were improved serum β-carotene levels in Zambian pre-school children that routinely consumed dishes prepared with biofortified maize [55]. Other studies have found that the consumption of biofortified maize resulted in an effective conversion of PVA into vitamin A [56,57]. These results reinforce the hypothesis that PVA biofortified maize could be used as a sustainable and effective complementary strategy to address VAD.

The PVA carotenoid concentration of OFSP was much higher than that of the CFSP, which, as expected, had a nutritionally insignificant PVA concentration. Regarding CFSP, the PVA values obtained in the current study agree with values reported in the literature [58]. The high PVA carotenoid concentration of OFSP could contribute to reducing VAD in vulnerable population groups. Another strategy that could be investigated is combining OFSP with other commonly consumed food item/s to enhance the nutrient content of the dishes, including provitamin A as well as other micronutrients with a high prevalence of deficiency. However, this study did not investigate the nutritional composition of composite dishes in which the OFSP was combined with other commonly consumed food items, and, therefore, further investigations are required. 

As stated earlier, mineral deficiencies, especially iron and zinc deficiencies, are a serious health problem in sub-Saharan African countries, including South Africa, but, unfortunately, they are often unnoticed and are not routinely treated. In the current study, the total mineral content (ash) of the individual curries (bambara groundnut, chicken, and cabbage curry, separately) was higher than that of PVA-biofortified *phutu*, which implies that the mineral content would be increased if either of the three curries were combined with PVA maize *phutu.* Yet again, chicken curry was the best option to improve the mineral content. Biofortified crops, if consumed in the correct quantities, could improve the micronutrient status of the affected communities [59]. Unlike a study conducted by Pillay et al. which found that the concentration of iron lower in PVA-biofortified maize than the white maize [60], the current study found no significant difference in iron concentration between white maize and PVA-biofortified maize. Furthermore, the results of the current study indicate that, regarding iron concentration in the composite dishes, there would be no benefit in replacing white *phutu* with PVA-biofortified *phutu* in the composite dishes, with respect to the iron and zinc concentration (Table 7). However, curried chicken contains a higher iron content, thus implying that if it were consumed together with PVA-biofortified *phutu* this could improve the iron intake of vulnerable populations. However, as mentioned earlier, chicken is unaffordable to many. Therefore, the more affordable combination, *phutu* and bambara groundnut curry, could be an alternative to improve the protein and micronutrient content of human diets. However, it needs to be emphasised that there is an urgent need to test consumer acceptability of this composite dish as it is not commonly consumed, especially in South Africa. Further studies could also investigate the effect of combining iron biofortified beans with PVA biofortified *phutu*, on the iron concentration.

Although fibre and total mineral concentrations were higher in OFSP than CFSP, the protein content of the OFSP was lower (Table 2). The OFSP of this study was high in iron concentration but low in zinc concentration, compared to the CFSP (Table 8). The results suggest that the OFSP could be used to improve the iron content of dishes in which sweet potato is a major ingredient, and, thereby, contribute to alleviation of iron deficiency among target communities. The OFSP could be composited with locally available, affordable food item/s rich in zinc, to simultaneously address zinc deficiency. 

Although, the protein content of the OFSP of this study was lower than that of the CFSP, it was higher than the values reported by Sanoussi et al., who found that the protein content ranged from 2.03-4.19 g/100 g, DW in pale-dark orange sweet potato [61]. The results of the current study and previous studies indicate that OFSP generally has a low protein content, and there is a need to develop the OFSP further to increase its protein content. Despite its low overall protein content, the concentration of the essential amino acid lysine was higher in OFSP than in the CFSP (Table 5). The consumption of OFSP could contribute to improving the fibre and iron content in the diets of vulnerable individuals. It is noteworthy that the current study did not investigate the nutritional composition of composite dishes comprised of OFSP and other locally available, affordable other food item. Furthermore, consumer acceptability of OFSP should be investigated and, if necessary, improved to ensure that OFSP would be consumed by the target population groups.

This study investigated the effect of replacing white maize and CFSP with PVA-biofortified maize and OFSP on the nutritional composition of traditional dishes. With respect to the protein and lysine concentration, the results of the current study indicate that there would not be an advantage in replacing white maize with PVA-biofortified maize (Table 1). This result is obviously because the protein content in white maize was not significantly different from PVA-biofortified maize. In contrast, Pillay et al. found that PVA-biofortified maize had a higher protein concentration than white maize [60], whereas Oluba and Oredokun-Lache found that white maize had a significantly higher value than PVA-biofortified maize [62]. The reason for the differences seen in protein concentration could be attributed to genetic and/or environmental factors. However, this study did not develop PVA-biofortified maize to improve the protein content, but focused on whether the PVA carotenoid and micronutrient content was increased. This was the reason why our results also indicated that PVA-biofortified maize had a similar lysine concentration to white maize (Table 3).

The protein content of dishes containing PVA-biofortified maize could be increased by combining the biofortified maize with protein-rich food items. For example, in the current study, when PVA-maize was combined with chicken curry, the composite dish had a higher protein content compared to PVA-biofortified *phutu* alone. This was expected, because, generally an animal food product such as chicken contains a higher protein content and quality than plant products; this is confirmed by the results of the current study where the curried chicken had a higher lysine content than all three plants products, PVA-biofortified *phutu,* curried cabbage, and curried bambara groundnut. Thus, combining PVA *phutu* with chicken curry would result in an improved protein content of the composite dish. However, the challenge is that chicken is not affordable to a large proportion of population groups in South Africa, especially those living in rural areas of KZN, where poverty and food insecurity are prevalent [43,44,45]. Although the protein concentration of the two composite dishes PVA *phutu* and cabbage curry, and PVA *phutu* and bambara groundnut curry were not significantly different (Table 1), the protein concentration of the composite dish containing bambara groundnut curry was higher numerically. It is well known that legume grains generally contain higher concentrations of protein than leafy vegetables. Furthermore, legumes contain adequate concentrations of lysine and tryptophan, whereas cereal grains contain an adequate concentration of methionine, but lysine and tryptophan are limiting [63]. Combining maize with a food item that is higher in lysine and tryptophan would improve the protein quality of the diet [64]. The deviation from this norm observed in this study could be attributed to the statistically small sample size. Therefore, it is still recommended that PVA *phutu* is combined with bambara groundnut curry rather than cabbage curry to improve the lysine concentration, thus improving the overall protein quality of the composite dish. Composite dishes with an improved protein content would be highly beneficial, especially to individuals that suffer from protein-energy malnutrition. This condition is caused predominately by a deficiency in protein and energy and leads to several serious health conditions [64,65]. Providing an affordable composite dish that combines PVA-biofortified *phutu* and bambara groundnut would not only contribute to reducing VAD, but also malnutrition. The main challenge with incorporating bambara groundnut into the diet of vulnerable groups is that this legume is not a common food source in South African [53]. It seems there are no published studies on consumer acceptability of composite dishes in which PVA *phutu* is combined with other food items such as cabbage and bambara groundnut. Thus, further investigation of consumer acceptability of these composite dishes would be required. 

## 5. Conclusions

The introduction of biofortified crops could provide nutritious, affordable food sources whose consumption would contribute significantly to improving the vitamin A status of the vulnerable population groups. The biofortified crops could be composited with underutilised nutrient-dense indigenous crops, such as bambara groundnut, to further fortify the dishes with essential nutrients and protein. 

The present study indicates that replacing white maize with PVA-biofortified maize in all the three composite dishes studied resulted in an improved PVA carotenoid content. Although the PVA *phutu* and chicken curry is the ideal composite dish for improving the PVA carotenoid content and protein quality, the composite dish containing PVA *phutu* and bambara groundnut curry would be a more affordable alternative, as it also has a high PVA carotenoid content and would likely have both high protein quality and content, due to the complementary protein phenomenon. There was no significant difference in the iron and zinc concentration of all the three composite dishes containing PVA *phutu*. The results further indicate that bambara groundnut would be a suitable alternative food source for compositing with provitamin A (PVA)-biofortified maize to improve the nutritional value of the traditional dishes that normally contain white maize as the main ingredient. However, consumer acceptability of the composite dishes containing bambara groundnut should be investigated further as the legume is not familiar to most South Africans.

The orange-fleshed sweet potato (OFSP) had high PVA carotenoid, fibre and iron concentration and a lower protein concentration compared to the cream-fleshed sweet potato (CFSP). The OFSP has the potential for improving the vitamin A status of VAD-vulnerable population groups in South Africa, if consumed with another food item, especially a protein-rich food, such a composite dish, would contribute to combating both VAD and malnutrition.

Overall, the findings of the current study indicate that Provitamin A-biofortified *phutu* when combined with other foods, such as curried cabbage, chicken or bambara groundnut and OFSP have the potential to improve nutrient intake and dietary diversity of the rural population groups in KZN and other rural areas of South Africa. The proposed composite foods would be new to the target population groups, as such it is not known whether they would be acceptable to the consumers. Therefore, future studies should investigate the sensory acceptability and consumer perceptions of combining PVA-biofortified *phutu* with either cabbage, chicken and bambara groundnut and OFSP.

## Figures and Tables

**Table 1:** Proximate composition of uncooked and cooked food samples except for sweet potato.

Sample	Moisture	Protein	Fat	NDF ^b^	Total Mineral Content (Ash)
	(%)	(g/100 g, DW ^a^)	(g/100 g, DW)	(g/100 g, DW)	(mg/100 g, DW)
**Raw maize meal/flour**					
White maize flour (control)	9.88 ^c^ (0.59) ^d^	10.22 (0.21)	3.89 (0.61)	5.44 (6.62)	1.21 (0.12)
PVA ^e^-biofortified maize flour	9.20 (1.77)	8.68 (0.01)	2.97 (0.15)	13.53 (0.85)	1.35 (0.04)
**Individual dishes**					
White *phutu* (control)	4.88 (0.17)	9.71 (0.15)	2.48 (0.21)	9.65 (0.62)	5.10 (0.37)
PVA-biofortified *phutu*	4.91 (0.50)	8.74 (0.03)	2.70 (0.28)	17.45 (0.66)	2.64 (0.15)
Cabbage curry	15.48 (1.68)	13.04 (0.56)	20.56 (0.03)	28.64 (1.75)	11.19 (0.28)
Chicken curry	9.17 (1.18)	71.23 (9.43)	22.62 (3.03)	40.82 (17.47)	14.15 (0.74)
Bambara groundnut curry	8.00 (0.27)	17.00 (0.17)	12.13 (0.97)	29.21 (4.71)	8.00 (0.27)
**Composite dishes**					
White *phutu* and chicken curry	4.17 (0.00)	28.60 (2.59)	7.27 (0.13)	10.77 (1.43)	3.42 (0.24)
PVA *phutu* and chicken curry	4.38 (0.54)	22.86 (0.87)	8.87 (1.29)	15.94 (4.55)	3.46 (0.06)
White *phutu* and cabbage curry	6.83 (0.82)	10.10 (0.11)	9.19 (0.35)	18.95 (2.81)	4.18 (0.25)
PVA *phutu* and cabbage curry	6.70 (0.63)	9.00 (0.15)	9.46 (0.10)	18.63 (0.23)	4.63 (0.17)
White *phutu* and bambara groundnut curry	7.14 (1.44)	13.29 (0.15)	8.98 (0.38)	17.45 (0.10)	3.24 (0.09)
PVA *phutu* and bambara groundnut curry	4.51 (2.75)	12.86 (0.93)	8.00 (0.77)	26.63 (2.19)	3.45 (0.11)
***P*-value ^f^**	**0.036**	**0.017**	**0.019**	**0.029**	**0.017**

^a^ DW: dry weight basis; ^b^ NDF: Neutral detergent fibre; ^c^ Mean of duplicate values; ^d^ Standard deviation; ^e^ PVA: Provitamin A; ^f^ Kruskal Wallis test; Values in bold indicate *P* < 0.05.

**Table 2:** Nutritional composition of cooked sweet potato.

Sweet Potato	Moisture	Protein	Fat	NDF ^b^	Total Mineral Content (Ash)
	(%)	(g/100 g, DW ^a^)	(g/100 g, DW)	(g/100 g, DW)	(mg/100 g, DW)
Boiled CFSP ^c^	3.90 ^d^ (0.64) ^e^	6.38 (0.32)	0.50 (0.11)	4.14 (0.18)	3.28 (0.92)
Boiled OFSP ^f^	4.88 (0.17)	4.51 (0.30)	0.64 (0.17)	5.97 (0.25)	5.83 (1.45)
*P*-value ^g^	**< 0.05**	**< 0.05**	1.000	**< 0.05**	**< 0.05**

^a^ DW: dry weight basis; ^b^ NDF: Neutral detergent fibre; ^c^ CFSP: Cream-fleshed sweet potato; ^d^ Mean of duplicate values; ^e^ Standard deviation; ^f^ OFSP: Orange- fleshed sweet potato; ^g^ Mann-Whitney U test; Values in bold indicate *P* < 0.05.

**Table 3:** Essential amino acid composition of uncooked and cooked samples except sweet potato (g/100 g, DW ^a^).

Sample	Histidine	Threonine	Lysine	Methionine	Valine	Lsoleucine	Leucine	Phenylalanine
**Raw milled maize meal/flour**								
White maize flour (control)	0.27 ^b^ (0.04) ^c^	0.33 (0.04)	0.40 (0.03)	ND ^d^	0.38 (0.06)	0.58 (0.06)	0.78 (0.01)	0.81 (0.11)
PVA ^e^-biofortified maize flour	0.20 (0.03)	0.27 (0.04)	0.36 (0.01)	ND	0.35 (0.05)	0.59 (0.10)	0.71 (0.11)	0.71 (0.02)
**Individual dishes**								
White *phutu* (control)	0.28 (0.02)	0.35 (0.11)	0.45 (0.11)	0.06 (0.08)	0.40 (0.19)	0.66 (0.59)	0.81 (0.37)	0.91 (0.05)
PVAe-biofortified *phutu*	0.20 (0.01)	0.27 (0.03)	0.28 (0.00)	0.08 (0.00)	0.35 (0.01)	0.43 (0.06)	0.67 (0.10)	0.68 (0.08)
Cabbage curry	0.15 (0.05)	0.33 (0.06)	0.59 (0.06)	0.05 (0.03)	0.36 (0.04)	0.63 (0.24)	0.30 (0.08)	0.59 (0.07)
Chicken curry	2.01 (0.07)	3.98 (0.27)	11.81(0.67)	2.27 (0.25)	3.18 (0.39)	7.63 (0.68)	4.69 (0.17)	3.02 (0.13)
Bambara groundnut curry	0.44 (0.06)	0.63 (0.07)	1.80 (0.04)	0.13 (0.02)	0.78 (0.06)	1.56 (0.14)	0.97 (0.08)	1.40 (0.35)
**Composite dishes**								
White *phutu* and chicken curry	0.55 (0.13)	1.03 (0.30)	2.23 (0.73)	0.36 (0.12)	0.88 (0.15)	1.95 (0.30)	1.52 (0.40)	1.31 (0.10)
PVA *phutu* and chicken curry	0.42 (0.12)	0.83 (0.16)	1.85 (0.61)	0.34 (0.07)	0.69 (0.12)	1.40 (0.63)	1.13 (0.28)	1.07 (0.06)
White *phutu* and cabbage curry	0.21 (0.05)	0.34 (0.05)	0.45 (0.03)	0.05 (0.01)	0.43 (0.02)	0.85 (0.13)	0.71 (0.00)	0.77 (0.12)
PVA *phutu* and cabbage curry	0.17 (0.08)	0.26 (0.06)	0.27 (0.15)	ND	0.35 (0.10)	0.38 (0.12)	0.59 (0.16)	0.97 (0.11)
White *phutu* and bambara groundnut curry	0.32 (0.04)	0.36 (0.06)	0.69 (0.22)	0.09 (0.02)	0.48 (0.08)	1.03 (0.10)	0.79 (0.02)	1.17 (0.11)
PVA *phutu* and bambara groundnut curry	0.25 (0.03)	0.32 (0.10)	0.69 (0.20)	0.08 (0.01)	0.45 (0.13)	0.73 (0.03)	0.62 (0.07)	1.09 (0.13)
***P*-value ^f^**	**0.030**	0.072	**0.021**	0.065	0.080	0.050	0.053	**0.024**

^a^ DW: dry weight basis; ^b^ Mean of duplicate values; ^c^ Standard deviation; ^d^ ND: Not detected; ^e^ PVA: Provitamin A; ^f^ Kruskal Wallis test; Values in bold indicate *P* < 0.05.

**Table 4:** Non-essential amino acid composition of uncooked and cooked samples except for sweet potato (g/100 g, DW ^a^).

Sample	Serine	Arginine	Glycine	Aspartate	Glutamate	Alanine	Proline	Tyrosine
**Raw milled maize meal/flour**								
White maize flour (control)	0.60 ^b^ (0.01) ^c^	0.42 (0.01)	0.76 (0.01)	0.45 (0.04)	1.89 (0.03)	0.66 (0.01)	0.76 (0.00)	0.15 (0.21)
PVA ^d^-biofortified maize flour	0.53 (0.06)	0.25 (0.05)	0.67 (0.16)	0.40 (0.05)	1.72 (0.30)	0.65 (0.07)	0.71 (0.11)	0.10 (0.13)
**Individual dishes**								
White *phutu* (control)	0.65 (0.30)	0.34 (0.09)	0.77 (0.22)	0.64 (0.29)	2.06 (1.01)	0.74 (0.32)	0.85 (0.28)	0.12 (N/A ^e^)
PVA-biofortified *phutu*	0.48 (0.04)	0.24 (0.00)	0.66 (0.03)	0.47 (0.06)	1.64 (0.23)	0.58 (0.04)	0.68 (0.01)	0.24 (N/A)
Cabbage curry	0.52 (0.06)	0.38 (0.05)	0.71 (0.21)	0.71 (0.04)	2.84 (0.47)	0.48 (0.06)	0.48 (0.13)	ND ^f^
Chicken curry	4.29 (0.18)	4.09 (0.08)	7.49 (0.59)	6.92 (0.26)	14.67 (0.59)	5.19 (0.23)	2.64 (0.16)	2.27 (0.07)
Bambara groundnut curry	1.26 (0.18)	0.88 (0.08)	1.45 (0.24)	1.81 (0.16)	3.68 (0.23)	0.85 (0.04)	0.68 (0.15)	0.46 (0.04)
**Composite dishes**								
White *phutu* and chicken curry	1.30 (0.31)	1.02 (0.30)	2.34 (0.27)	1.62 (0.49)	4.37 (1.21)	1.62 (0.32)	1.15 (0.12)	0.53 (0.14)
PVA *phutu* and chicken curry	1.08 (0.20)	0.78 (0.14)	1.79 (0.28)	1.26 (0.33)	3.48 (0.98)	1.32 (0.23)	0.96 (0.16)	ND
White *phutu* and cabbage curry	0.65 (0.01)	0.27 (0.05)	0.77 (0.01)	0.57 (0.02)	2.28 (0.12)	0.71 (0.06)	0.77 (0.01)	ND
PVA *phutu* and cabbage curry	0.52 (0.12)	0.24 (0.13)	0.63 (0.16)	0.50 (0.18)	1.99 (0.62)	0.61 (0.17)	0.67 (0.14)	ND
White *phutu* and bambara groundnut curry	0.75 (0.09)	0.47 (0.07)	0.96 (0.04)	0.83 (0.12)	2.22 (0.47)	0.71 (0.04)	0.69 (0.01)	0.32 (0.02)
PVA *phutu* and bambara groundnut curry	0.76 (0.20)	0.42 (0.16)	0.83 (0.22)	0.89 (0.15)	1.90 (0.42)	0.61 (0.09)	0.57 (0.04)	0.22 (0.02)
***P*-value ^g^**	**0.043**	**0.032**	0.063	**0.032**	0.068	0.074	0.088	0.088

^a^ DW: dry weight basis; ^b^ Mean of duplicate values; ^c^ Standard deviation; ^d^ PVA: Provitamin A; ^e^ N/A: Not applicable; ^f^ ND: Not detected; ^g^ Kruskal Wallis test; Values in bold indicate *P* < 0.05.

**Table 5:** Essential amino acid composition of sweet potato (g/100 g, DW ^a^).

Sweet Potato	Histidine	Threonine	Lysine	Methionine	Valine	Lsoleucine	Leucine	Phenylalanine
Boiled CFSP ^b^	ND ^c^	0.13 ^d^ (0.03) ^e^	0.24 (0.04)	ND	0.20 (0.05)	0.14 (0.04)	0.08 (0.01)	0.66 (0.03)
Boiled OFSP ^f^	0.05 (0.01)	0.19 (0.06)	0.38 (0.04)	ND	0.20 (0.06)	0.26 (0.02)	0.16 (0.04)	0.32 (0.03)
*P*-value ^g^		0.500	**< 0.05**		1.500	**< 0.05**	**< 0.05**	**< 0.05**

^a^ DW: dry weight basis; ^b^ CFSP: Cream-fleshed sweet potato; ^c^ ND: Not detected; ^d^ Mean of duplicate values; ^e^ Standard deviation; ^f^ OFSP: Orange-fleshed sweet potato; ^g^ Mann-Whitney U test; Values in bold indicate *P* < 0.05.

**Table 6:** Non-essential amino acid composition of sweet potato (g/100 g, DW ^a^).

Sweet Potato	Serine	Arginine	Glycine	Aspartate	Glutamate	Alanine	Proline	Tyrosine
Boiled CFSP ^b^	0.30 ^c^ (0.04) ^d^	0.09 (0.02)	0.34 (0.07)	0.48 (0.08)	0.35 (0.06)	0.26 (0.01)	0.09 (0.01)	ND ^e^
Boiled OFSP ^f^	0.33 (0.10)	0.12 (0.03)	0.39 (0.10)	0.77 (0.15)	0.59 (0.11)	0.30 (0.03)	0.13 (0.04)	0.04 (N/A ^g^)
*P*-value ^h^	2.000	0.500	1.000	**< 0.05**	**< 0.05**	**< 0.05**	0.500	

^a^ DW: dry weight basis; ^b^ CFSP: Cream-fleshed sweet potato; ^c^ Mean of duplicate values; ^d^ Standard deviation; ^e^ ND: Not detected; ^f^ OFSP: Orange-fleshed sweet potato; ^g^ N/A: Not applicable; ^h^ Mann-Whitney U test; Values in bold indicate *P* < 0.05.

**Table 7:** Concentration of individual mineral elements of uncooked and cooked food samples except sweet potato (mg/100 g, DW ^a^).

Sample	Calcium	Magnesium	Potassium	Sodium	Phosphorous	Zinc	Iron
**Raw milled maize meal/flour**							
White maize flour (control)	0.00 ^b^(0.00) ^c^	0.11 (0.01)	0.31 (0.01)	0.00 (0.00)	0.26 (0.01)	1.80 (0.14)	2.05 (2.21)
PVA ^d^-biofortified maize flour	0.01 (0.01)	0.11 (0.00)	0.33 (0.01)	0.00 (0.00)	0.27 (0.01)	1.80 (0.14)	2.10 (0.28)
**Individual dishes**							
White *phutu* (control)	0.01 (0.00)	0.10 (0.00)	0.36 (0.01)	1.73 (0.02)	0.25 (0.01)	1.55 (0.07)	1.60 (0.00)
PVA biofortified *phutu*	0.01 (0.00)	0.11 (0.01)	0.34 (0.01)	0.60 (0.00)	0.26 (0.01)	1.70 (0.00)	1.70 (0.42)
Cabbage curry	0.35 (0.00)	0.15 (0.00)	1.56 (0.01)	2.75 (0.02)	0.27 (0.00)	1.60 (0.14)	3.25 (0.07)
Chicken curry	1.52 (0.67)	1.90 (0.10)	29.20 (2.44)	28.02 (3.92)	13.82 (0.81)	3259.10 (1450.70)	98.50 (39.46)
Bambara groundnut curry	0.04 (0.00)	0.15 (0.01)	1.60 (0.45)	2.00 (0.11)	0.25 (0.00)	1.90 (0.14)	1.90 (0.00)
**Composite dishes**							
White *phutu* and chicken curry	0.10 (0.03)	0.11 (0.01)	0.60 (0.01)	0.71 (0.00)	0.38 (0.03)	1.80 (0.00)	2.15 (0.07)
PVA *phutu* and chicken curry	0.03 (0.00)	0.10 (0.00)	0.59 (0.05)	0.75 (0.03)	0.36 (0.00)	2.40 (0.42)	2.75 (0.07)
White *phutu* and cabbage curry	0.08 (0.00)	0.11 (0.01)	0.56 (0.01)	0.92 (0.00)	0.25 (0.01)	2.15 (0.64)	2.65 (0.21)
PVA *phutu* and cabbage curry	0.10 (0.01)	0.11 (0.01)	0.58 (0.01)	1.07 (0.04)	0.23 (0.00)	1.70 (0.14)	2.25 (0.21)
White *phutu* and bambara groundnut curry	0.02 (0.00)	0.13 (0.00)	0.78 (0.01)	0.44 (0.00)	0.28 (0.01)	2.05 (0.21)	2.15 (0.07)
PVA *phutu* and bambara groundnut curry	0.02 (0.00)	0.12 (0.00)	0.73 (0.04)	0.47 (0.02)	0.26 (0.00)	1.80 (0.00)	2.10 (0.14)
*P*-value ^e^	**0.016**	**0.037**	**0.018**	**0.015**	**0.024**	0.085	**0.042**

^a^ DW: dry weight basis; ^b^ Mean of duplicate values; ^c^ Standard deviation; ^d^ PVA: Provitamin A; ^e^ Kruskal Wallis test; Values in bold indicate *P* < 0.05.

**Table 8:** Concentration of individual mineral elements of cooked sweet potato (mg/100 g, DW ^a^).

Sweet Potato	Calcium	Magnesium	Potassium	Sodium	Phosphorous	Zinc	Iron
Boiled CFSP ^b^	0.13 ^c^ (0.02) ^d^	0.13 (0.01)	1.35 (0.03)	0.07 (0.01)	0.21 (0.00)	1.30 (0.14)	2.25 (0.21)
Boiled OFSP ^e^	0.06 (0.00)	0.09 (0.00)	1.70 (0.01)	0.03 (0.01)	0.15 (0.00)	0.45 (0.64)	2.55 (0.07)
*P*-value ^f^	**< 0.05**	**< 0.05**	**< 0.05**	**< 0.05**	**< 0.05**	**< 0.05**	**< 0.05**

^a^ DW: dry weight basis; ^b^ CFSP: Cream-fleshed sweet potato; ^c^ Mean of duplicate values; ^d^ Standard deviation; ^e^ OFSP: Orange-fleshed sweet potato; ^f^ Mann- Whitney U test; Values in bold indicate *P* < 0.05.

**Table 9:** Provitamin A content of provitamin A-biofortified maize composite dishes (µg/g, DW ^a^).

Sample	Zeaxanthin	β-Cryptoxanthin	Lutein	β-Carotene Isomers			Total β-Carotene ^b^	Provitamin A Carotenoids ^c^	Total Carotenoids ^d^
				β-Carotene	9-cis	13-cis			
**Raw milled maize meal/flour**									
White maize flour (control)	0.27 ^e^ (0.02) ^f^	0.00 (0.00)	0.03 (0.00)	0.22 (0.01)	0.20 (0.01)	0.20 (0.01)	0.62 (0.04)	0.62 (0.04)	0.92 (0.06)
PVA ^g^-biofortified maize flour	6.35 (0.46)	0.53(0.42)	1.12 (0.08)	0.51 (0.04)	0.36 (0.03)	0.29 (0.02)	1.16 (0.09)	1.43 (0.11)	9.15 (0.67)
**Individual dishes**									
White *phutu* (control)	0.20 (0.01)	0.00 (0.00)	0.03 (0.00)	0.21 (0.01)	0.20 (0.01)	0.19 (0.01)	0.60 (0.04)	0.60 (0.04)	0.83 (0.05)
PVA-biofortified *phutu*	2.44 (0.16)	0.51 (0.04)	0.74 (0.04)	0.47 (0.04)	0.35 (0.03)	0.26 (0.01)	1.08 (0.08)	1.34 (0.09)	4.76 (0.32)
Cabbage curry	0.92 (0.09)	0.10 (0.14)	0.16 (0.02)	0.43 (0.04)	0.26 (0.02)	0.23 (0.03)	0.92 (0.09)	0.97 (0.11)	2.09 (0.22)
Chicken curry	0.70 (0.04)	0.00 (0.00)	0.03 (0.00)	0.21 (0.01)	0.00 (0.00)	0.00 (0.00)	0.21 (0.01)	0.21 (0.01)	0.94 (0.05)
Bambara groundnut curry	0.20 (0.01)	0.00 (0.00)	0.03 (0.00)	0.23 (0.01)	0.20 (0.01)	0.20 (0.01)	0.63 (0.04)	0.63 (0.04)	0.85 (0.04)
**Composite dishes**									
White *phutu* and chicken curry	0.50 (0.04)	0.00 (0.00)	0.14 (0.01)	0.20 (0.01)	0.20 (0.01)	0.18 (0.05)	0.58 (0.08)	0.60 (0.05)	1.21 (0.12)
PVA *phutu* and chicken curry	1.35 (0.09)	0.35 (0.02)	0.43 (0.28)	0.39 (0.03)	0.32 (0.02)	0.24 (0.01)	0.95 (0.06)	1.12 (0.07)	3.07 (0.21)
White *phutu* and cabbage curry	1.25 (0.08)	0.00 (0.00)	0.28 (0.02)	0.20 (0.01)	0.20 (0.01)	0.20 (0.01)	0.60 (0.04)	0.60 (0.04)	2.12 (0.14)
PVA *phutu* and cabbage curry	0.81 (0.06)	0.14 (0.01)	0.17 (0.01)	0.27 (0.02)	0.23 (0.01)	0.21(0.01)	0.71 (0.05)	0.78 (0.06)	1.83 (0.13)
White *phutu* and bambara groundnut curry	0.16 (0.01)	0.00 (0.00)	0.03 (0.00)	0.20 (0.01)	0.21 (0.01)	0.20 (0.01)	0.61 (0.04)	0.61 (0.04)	0.79 (0.04)
PVA *phutu* and bambara groundnut curry	1.02 (0.06)	0.25 (0.01)	0.30 (0.02)	0.34 (0.02)	0.28 (0.01)	0.23 (0.01)	0.85 (0.05)	0.97 (0.06)	2.41 (0.15)
*P*-value ^h^	**0.016**	**0.015**	**0.016**	**0.028**	**0.028**	0.055	**0.029**	**0.028**	**0.017**

^a^ DW: dry weight basis; ^b^ (β-carotene + 9-cis +13-cis); ^c^ (β-cryptoxanthin/2 + β-carotene + 9-cis + 13-cis); ^d^ (Total β carotene + Zeaxanthin, + β-cryptoxanthin + Lutein); ^e^ Mean of duplicate values; ^f^ Standard deviation; ^g^ PVA: Provitamin A; ^h^ Kruskal Wallis test; Values in bold indicate *P* < 0.05.

**Table 10:** Provitamin A content of provitamin A-biofortified sweet potato (µg/g, DW ^a^).

Sweet Potato	Zeaxanthin	β-Cryptoxanthin	Lutein	β-Carotene Isomers			Total β-Carotene ^b^	Provitamin A Carotenoids ^c^	Total Carotenoids ^d^
				β-Carotene	9-*cis*	13-*cis*			
Boiled CFSP ^e^	0.20 ^f^ (0.01) ^g^	0.10 (0.01)	0.03 (0.00)	0.27 (0.01)	0.22 (0.01)	0.23 (0.01)	0.72 (0.04)	0.77 (0.04)	1.05 (0.06)
Boiled OFSP ^h^	0.40 (0.02)	0.35 (0.14)	0.05 (0.01)	43.29 (1.99)	3.18 (0.15)	9.20 (0.42)	55.67 (2.57)	55.84 (2.57)	56.46 (2.61)
*P*-value ^i^	**< 0.05**	**< 0.05**	**< 0.05**	**< 0.05**	**< 0.05**	**< 0.05**	**< 0.05**	**< 0.05**	**< 0.05**

^a^ DW: dry weight basis; ^b^ (β-carotene + 9-cis +13-cis); ^c^ (β-cryptoxanthin/2 + β-carotene + 9-cis + 13-cis); ^d^ (Total β carotene + Zeaxanthin, + β-cryptoxanthin + Lutein); ^e^ CFSP: Cream-fleshed sweet potato; ^f^ Mean of duplicate values; ^g^ Standard deviation; ^h^ OFSP: Orange-fleshed sweet potato; **^i^** Mann-Whitney U test; Values in bold indicate *P* < 0.05.

## Supplementary Materials

The following are available online at https://www.mdpi.com/2072-6643/11/6/1198/s1, S1: Standardised recipe for the preparation of *phutu*, S2: Standardised recipe for the preparation of cabbage curry, S3: Standardised recipe for the preparation of chicken curry, S4: Standardised recipe for the preparation of bambara groundnut curry, S5: Standardised recipe for the preparation of sweet potato.
