# Supplementary material for: Improving the Dietary Vitamin A Content of Rural Communities in South Africa by Replacing Non-Biofortified White Maize and Sweet Potato with Biofortified Maize and Sweet Potato in Traditional Dishes
Source: Nutrients. 2019 May 28;11(6):1198. doi: 10.3390/nu11061198 (PMC6628247; doi:10.3390/nu11061198)
Supplement: Supplementary file 1 [file nutrients-11-01198-s001.pdf]

S1: Standardised recipe for the preparation of *phutu*

Ingredients
625 mL water
1 mL salt
4 cups (536 g) mealie meal [White maize/ Provitamin A-biofortified maize]

Method:
1. Bring 625 mL of water to the boil in a heavy-bottom pot on a Defy Thermofan Stove (Model 731 MF) on high heat (plate control setting 6).
2. Add 1 mL of salt to the water.
3. Add 4 cups of mealie meal (536 g) to the boiling water and stir as soon as the mixture reaches boiling point.
4. Allow the *phutu* to stand on low heat (plate control setting 1) for approximately 75 minutes, with the pot lid on and occasional stirring until cooked.

S2: Standardised recipe for the preparation of cabbage curry

Ingredients
2 medium cabbage (Shred cabbage)
1 finely chopped medium onion
2 tsp Raja curry spice
1 tsp salt
1 ladle spoon of sunflower oil
1 x Knorrox cube (oxtail spice)
400 mL water

Method:
1. Heat 1 ladle spoon sunflower oil in a heavy-bottom pot on a Defy Thermofan Stove (Model 731 MF) on medium heat (plate control setting 4).
2. Add 2 teaspoons Raja curry spice and finely chopped onions to the hot oil. Allow to brown.
3. Once the onions have browned, add the shredded cabbage and 400 mL water and allow the cabbage to cook with the pot lid on and occasional stirring.
4. When the cabbage has softened, add 1 teaspoon salt and break 1 Knorrox cube into the pot.
5. Allow the cabbage curry to continue cooking on low heat (plate control setting 2) for approximately 20 minutes with the pot lid on and occasional stirring until cooked.

S3: Standardised recipe for the preparation of chicken curry

Ingredients
3kg of cut chicken pieces
2 finely chopped medium onions
2 tsp Raja curry spice
1 tsp salt
1 ladle spoon of sunflower oil
2 x Knorrox cube (oxtail spice)
250 mL water

Method
1. Heat 1 ladle spoon of sunflower oil in a heavy-bottom pot on a Defy Thermofan Stove (Model 731 MF) on medium heat (plate control setting 4).
2. Add 2 teaspoons Raja curry spice and finely chopped onions to the hot oil. Allow to brown.
3. Once the onions have browned, add cut chicken pieces and 250 mL of water and allow the chicken to cook with the pot lid on and occasional stirring.
4. Half an hour later add the 1 teaspoon of salt and 2 knorrox cubes into the pot.
5. Allow the chicken curry to continue cooking on low heat (plate control setting 2) for approximately 30 minutes with the pot lid on and occasional stirring until cooked.

S4: Standardised recipe for the preparation of bambara groundnut curry

Ingredients

4 cups of bambara groundnut (Soaked overnight)
16 cups of water (4000 mL)
2 finely chopped medium onions
1 tsp Raja curry spice
1 tsp salt
1 tsp bicarbonate of soda
2 ladle spoons of sunflower oil
1 x Knorrox cube (oxtail spice)

Method:

1. Bring 2000 mL of water to the boil in a heavy-bottom pot on a Defy Thermofan Stove (Model 731 MF) on high heat (plate control setting 6) and add bambara groundnut.
2. Half an hour later, add 2 ladle spoons of sunflower oil and 2 finely chopped onions. Allow the bambara groundnut to continue cooking on medium heat (plate control setting 4).
3. When the water starts to reduce, add 1 mL bicarbonate of soda and 1000mL water. Allow the bambara groundnut curry to continue cooking on medium heat (plate control setting 4).
4. When the bambara groundnut becomes soft, add 1 teaspoon of Raja curry spice, 1 teaspoon of salt and 1 knorrox cube.
5. Add a further 1000mL of water and allow the bambara groundnut curry to continue cooking on low heat (plate control setting 2) for approximately 40 minutes with the pot lid on and occasional stirring until cooked.

S5: Standardised recipe for the preparation of sweet potato

Ingredients
5kg sweet potato
12 cups of water (3000 mL)

Method
1. Bring 3000 mL of water to the boil in a heavy-bottom pot on a Defy Thermofan Stove (Model 731 MF) on high heat (plate control setting 6).
2. Add sweet potato to the boiling water.
3. Allow the sweet potato to continue boiling on medium heat (plate control setting 4) for approximately 45 minutes.
